# Palearctic flea beetle and pest of hops and *Cannabis*, *Psylliodesattenuata* (Coleoptera, Chrysomelidae, Galerucinae), new to North America

**DOI:** 10.3897/BDJ.12.e120340

**Published:** 2024-07-05

**Authors:** Hume B Douglas, Justin Renkema, Tyler W Smith, Alexander S Konstantinov, Joseph Moisan-De Serres

**Affiliations:** 1 Agriculture and Agri-Food Canada, Ottawa, Canada Agriculture and Agri-Food Canada Ottawa Canada; 2 Agriculture and Agri-Food Canada, Vineland, Canada Agriculture and Agri-Food Canada Vineland Canada; 3 Systematic Entomology Laboratory, USDA, ARS, Smithsonian Institution, National Museum of Natural History, MRC-168 Washington, United States of America Systematic Entomology Laboratory, USDA, ARS, Smithsonian Institution National Museum of Natural History, MRC-168 Washington United States of America; 4 Ministère de l'Agriculture, des Pêcheries et de l'Alimentation du Québec, Québec, Canada Ministère de l'Agriculture, des Pêcheries et de l'Alimentation du Québec Québec Canada

**Keywords:** invasive alien species, adventive species, crop pest, faunal record, flea beetle, hops, hemp, *Humulus*, *Cannabis*

## Abstract

**Background:**

The univoltine leaf beetle *Psylliodesattenuata* (Koch, 1803) is a pest of *Cannabis* and *Humulus* (Cannabaceae) and native to the Palaearctic Region, known from eastern Asia to western Europe.

**New information:**

First North American records are presented for *P.attenuata* from Canada: Ontario and Québec. Adult beetle feeding damage to hops *Humuluslupulus* L. (Cannabacaea) plants is recorded from Québec. Diagnostic information is presented to distinguish *P.attenuata* from other North American Chrysomelidae and a preliminary assessment of its potential to spread in North America is presented. While our climate analysis is limited by a lack of data, it appears *P.attenuata* is physiologically capable of persisting throughout the range of *Humulus* in North America.

The United States of America and Canada are now known to be home to 71 or more species of adventive Chrysomelidae.

## Introduction

The univoltine leaf beetle *Psylliodesattenuata* (Koch, 1803) is native to the Palaearctic Region from eastern Asia to western Europe (Döberl 2010). It is a widespread and common species in some countries and rare in others (Rheinheimer and Hassler 2018), breeding on Cannabaceae: *Cannabis* L. and *Humulus* L. (Rheinheimer and Hassler 2018). Joseph Moisan-De Serres (co-author) first became aware of flea beetle damage to *Humuluslupulus* L (hops) plants in Québec Canada in 2022 when he was contacted by an agronomist scouting hop yards (hops plantations). Justin Renkema (co-author) encountered the same populations in Québec the same year and in additional populations in Ontario, while sampling mites and arthropod diversity at hop yards. We aimed to identify these beetles and establish whether this species is present in hop-growing areas of Québec and Ontario and to complete a preliminary assessment of its potential spread by contrasting climatic conditions in its native range with those in North America.

## Materials and methods

An agronomist working in hop yards contacted Joseph Moisan-De Serres (JM-D) in 2022 to investigate flea beetle damage to hop yards (hop yards) near Québec City. JM-D collected specimens, recognised these as *Psylliodesattenuata* and submitted them to HD for dissection and further identification. Justin Renkema (JR) was simultaneously conducting a survey for foliar arthropod diversity at four hop yards in southern Québec and five in Ontario by shaking bines (stems) over a 1 m^2^ funnel and also submitted *Psylliodes* samples to HD. HD dissected specimens and compared them to North American and Palaearctic literature, also sending photographs to Alexander Konstantinov (AK) for confirmation. Specimens are deposited in the Canadian National Collection of Insects Arachnids and Nematodes, Ottawa Ontario, Canada. Specimen occurrences were mapped using SimpleMappr (Shorthouse 2010).

For DNA analysis, three specimens were sent to the Centre for Biodiversity Genomics (CBG, University of Guelph, Guelph, Ontario, Canada). There, a single leg was detached from each specimen and was placed in a well in a 96-well microplate prefilled with 10 µl of 96% ethanol. Each specimen was also photographed and the resulting image was uploaded to the Barcode of Life Database (BOLD; Ratnasingham and Hebert (2013)) along with the label data. The DNA extraction, polymerase chain reaction amplification and Sanger sequencing of the cytochrome oxidase subunit 1 barcode region were performed for all specimens at the CBG, using standard protocols as outlined by Pentinsaari et al. (2019). Primers *C_LepFoIF* and *C_LepFoIR* (Woodcock et al. 2013) were used for polymerase chain reaction amplification. Sequences were obtained through unidirectional analysis. Details on the polymerase chain reaction and sequencing protocols for each specimen are provided in the public BOLD dataset information below.

Detailed collection information for each specimen, including both DNA-barcoded material and other specimen records, as well as GenBank accession numbers for the barcode sequences, are listed in the text. All sequences, details on polymerase chain reaction and sequencing primers, photographs and full collection data for the DNA-barcoded specimens are available through a public dataset on BOLD (https://doi.org/dx.doi.org/10.5883/ DS-PAQ2023).

As an assessment of the potential distribution of *P.attenuata* in North America, TWS initially intended to prepare a species distribution model using the programme Maxent (Phillips et al. 2017). However, after downloading all GBIF records (n = 242, GBIF.Org (2023)) it was apparent that these data capture only a small portion of the beetle's western and south-eastern range, while most of its wide distribution in eastern Europe and Asia (Döberl 2010) is unsampled. In the absence of adequate occurrence data for robust modelling, we elected to use a simpler approach. We compared the known *P.attenuata* records to the distribution of its host plant *Humulus* spp. We did not include *Cannabis* records in this assessment, as the long history of illicit cultivation of this species makes it difficult to know with certainty what its actual range in the wild is. However, we did review GBIF records for *Cannabis* and the northern limit of known collections coincides closely with the northern limit for *Humulus* in North America (data not shown). Additionally, we determined the minimum winter temperature corresponding to the documented *P.attenuata* records, as a preliminary assessment of its cold tolerance.

We downloaded all *Humulus* L. records with geographic coordinates from GBIF (n = 310323, GBIF.Org (2024)). The majority of these records (n = 298,068) were identified as *Humuluslupulus* L. and did not indicate a variety. Thus, we could not distinguish amongst taxa native to North America (i.e. H.lupulusvar.neomexicanus A. Nelson & Cockerell, H.lupulusvar.pubescens E. Small and H.lupulusvar.lupuloides E. Small) and the introduced H.lupulusvar.lupulus. The latter is the main ancestor of the cultivated crop, although all varieties have been incorporated to some extent in modern cultivars (Small 2016a).

The second most numerous taxon (n = 10353) was *H.scandens* (Lour.) Merr., an annual plant native to Eurasia, often referred to as *H.japonicus* Siebold & Zuccarini (Small 1997). As *P.attenuata* can feed on all of these taxa, we did not distinguish amongst them in our map.

To estimate the winter cold tolerance of *P.attenuata*, we extracted the coldest temperature from the coldest month for each record, using the WorldClim global climate data (Bioclim Variable 6; Fick and Hijmans (2017)). We then constructed an isotherm for this value, to visualise the geographic area where cold tolerance is presumably not a limiting factor for this species.

## Data resources

The new occurrence data reported in this paper are deposited at GBIF, the Global Biodiversity Information Facility: https://doi.org/10.15468/p3h5k2

## Taxon treatments

### Psylliodes (Psylliodes) attenuata

(Koch, 1803)

38CA33CC-7899-5F0B-83AF-F366CE6AD82F

https://www.gbif.org/species/7382301

#### Diagnosis

*Psylliodesattenuata* is 1.6-2.6 mm long (Figs 1, 2) and can be recognised as belonging to genus *Psylliodes* by its jumping hind legs and antennae with ten antennomeres (Fig. 1a, b). It can be distinguished from other North American *Psylliodes* by its green-bronze dorsal colouration (translucent and paler at elytral apices); head with antennal calli present and defined by grooves posterad (Fig. 1c, forming an ‘x’ together with grooves below calli), without puncture-like central pit at centre of the ‘x’. Additionally, male only characters which can be used for confirmation include: protarsus with basal segment widest before apex (Fig. 1e, apical 3/4); abdominal ventrite V with median quarter of posterior edge elongate (Fig. 1d) and flexed dorsally posterad; aedeagus widest at basal third with apex attenuate (Fig. 2b).

#### Distribution

**Figure 3.**
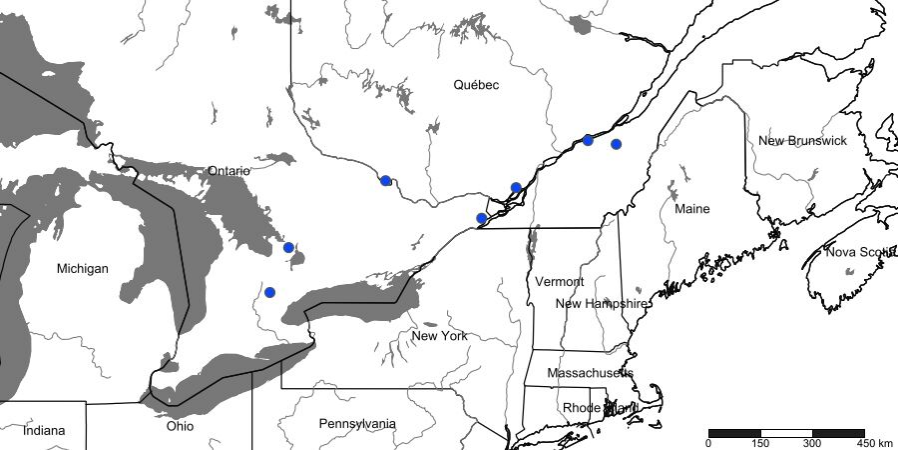
Map of records of *Psylliodesattenuata* from eastern Canada.

We recorded *Psylliodesattenuata* from the eight illustrated localities in Canada (Fig. 3). Specimen data are available via GBIF.org in Douglas (2024). Surveys further to the southwest in Ontario by JR at hop yards near Tavistock (Wellington County) and Aylmer (Elgin County) did not result in captures of *P.attenuata* specimens.

#### Biology

The finding of 752 specimens from eight sites separated by at least 750 km indicates that multiple breeding populations of *P.attenuata* are established in Canada. Like many flea beetles, *P.attenuata* are univoltine, with egg laying and below-ground development of immatures during summer. New adults emerge in August and September in UK and then overwinter in vegetation, litter and soil (Cox 2007). Adults are fully winged and have been collected in UK from March to September, with most in May, August and September (Cox 2007).

##### Feeding damage to hop plants

We observed substantial damage to some of the assessed hop yards (Fig. 4). Growers reported that new hop shoots were severely defoliated by overwintering adult beetles in May, stunting growth for up to 1 week on some varieties. Given the rapid growth of the plants and the departure of the overwintering adults during June, damage became less noticeable during July. Additional damage became evident in mid-August following the emergence of a new cohort of adult beetles. Damage levels increased up to harvest time. Beetles attacked mainly lateral shoots, flowers and fruiting bodies (cones) during August and September. In some hop yards, approximately one in three cones contained a feeding flea beetle. Cones with extensive feeding damage desiccated and turned brown (Fig. 4f). This appeared similar to symptoms of caused by downy mildew (*Podosphaeramacularis*), except that beetle-feeding also causes holes in plant tissue. Growers estimated yield losses of at least at 10-15% in heavily infested hop yards.

#### Notes

The external morphology and male genitalia of the Canadian specimens closely matched taxon concepts of *P.attenuata*. Specimens were confirmed as *P.attenuata* using Mohr (1966) and Rheinheimer and Hassler (2018) and in comparison with specimens from the Canadian National Collection of Insects Arachnids and Nematodes (CNCI) identified by L.H. Woollat (UK) and M. Döberl (Germany) and the United States National Museum (USNM) identified by F. Heikertinger. As the only bronze-coloured *Psylliodes* with a complete ‘x’ shaped impression on the frons (Fig. 1c,Fig. 1a, b) and no frontal pit, *P.attenuata* is unlikely to be confused with any other North American species.

##### DNA Barcoding Results

Analysis of the two DNA-barcoded Canadian specimens of *Psylliodes* through the BOLD Identification Engine resulted in a 100% match with *P.attenuata* for one specimen. This specimen shared a BOLD Barcode Index Number (BIN, Ratnasingham and Hebert (2013)) with a specimen from Germany (BIN ACJ8391) and the other formed its own neighbouring BIN (AFE7531). Comparison of sequences of our specimens to the available *Psylliodes* specimens from barcoding initiatives (Hendrich et al. 2014, Pentinsaari et al. 2014) indicated sequence divergences of 0–3.5% amongst 60 public and non-public records in BIN ACJ8391. The morphological identification of these specimens as *P.attenuata*, their presence on hops plants and the finding that one of two DNA barcoded specimens exactly matched the sequence of a specimen from Germany all support the conclusion that the Canadian specimens are *P.attenuata*.

## Analysis

### Potential distribution

**Figure 5.**
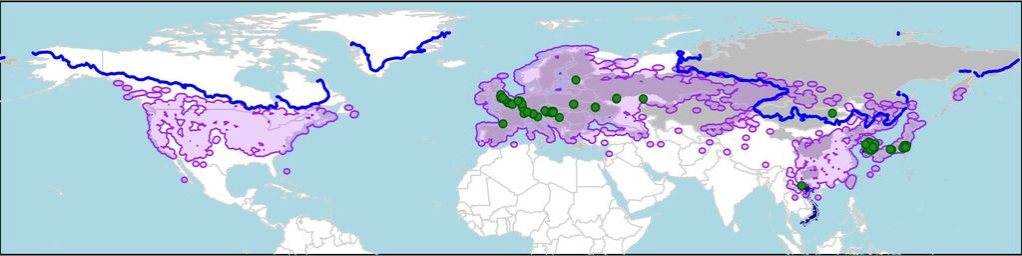
Distribution of *Psylliodesattenuata* and its *Humulus* spp. host plant. Jurisdictions where *P.attenuata* has been documented are indicated by grey (Döberl 2010) and blue shading (Vietnam and South Korea, records from GBIF and CNC). Green points indicate georeferenced *P.attenuata* specimens. Purple shading indicates the range of *Humulus* spp. in North America and Eurasia, based on GBIF records. The blue line is the approximate -25.5°C minimum isotherm, which corresponds to the coldest average monthly temperature for any of the *P.attenuata* records.

A record from Mongolia produced the lowest winter temperature value, -25.5°C. The isotherm for this value closely follows the northern limit of *Humulus* in Eurasia and matches the distribution of *Humulus* in North America almost exactly (Fig. 5). This may indicate the actual physiological cold tolerance limit for *P.attenuata* or it may reflect the cold hardiness of *Humulus* limiting the availability of host plants north of this line. In either case, it suggests *P.attenuata* is physiologically capable of establishing throughout the northern range of *Humulus* in North America.

## Discussion

### Adventive species biology

Adult *P.attenuata* feed on *Humulus*, *Cannabis* and *Urtica* L. (Urticaceae) in their native range. Larvae develop on and in roots and root crowns of *Humulus* and *Cannabis* (Rheinheimer and Hassler 2018). Like most flea beetles, we expect adults feeding on shoots, leaves and inflorescences to cause the greatest damage.

Given that this species was first detected as a pest of hops, it can be expected to act as a pest in hop yards in North America and possibly to feed on wild hops plants. There are three *Humuluslupulus* varieties native to North America: var. neomexicanus, var. pubescens and var. lupuloides. Collectively, they are present in all Canadian provinces and all US states other than the southeast and Pacific northwest (Small 1997). Additionally, H.lupulusvar.lupulus has been introduced as a crop and persists around abandoned homesteads, occasionally hybridising with the native varieties (Small 2016a). Commercial production is concentrated in Oregon, Idaho and Washington State, where 50-60 thousand acres have been cultivated in recent years, with a production value of $560-$660 million (US dollars; National Agricultural Statistics Service (2023)). *Humulusscandens* has also been introduced as an ornamental, with weedy populations across eastern North America. It does not have any agricultural value.

*Psylliodesattenuata* can also be expected to feed on outdoor *Cannabis* plants in North America. *Cannabis* has been documented growing wild across North America, although it is difficult to distinguish self-sustaining populations from short-lived escapes from (legal or illicit) plantings (Small 2016b). *Cannabis* is cultivated as hemp and marijuana, depending on the concentration of the psychoactive tetrahydrocannabinol (THC). Hemp, with THC < 0.3%, is widely grown, with 13 k hectares cultivated in Canada (Health Canada 2024) and 22 k in the US (National Agricultural Statistics Service 2022). A further 685 hectares of marijuana (THC > 0.3%) were cultivated outdoors in Canada in 2021 (mostly in British Columbia, Statistics Canada (2021)). We could not find outdoor acrages for the USA, but the combined value of greenhouse and outdoor crops was more than $5B US dollars in 2022 according to an industry association (Downs and Williams 2023).

*Urtica* is common across Canada and the USA, particularly two native subspecies of *U.dioica* L. (Boufford 1997). While *P.attenuata* cannot complete its life cycle on these plants, they could facilitate its dispersal amongst *Humulus* and *Cannabis* populations. Cox (2007) recorded several species of the parasitoid wasp *Perilitus* Nees, 1818 (Hymenoptera, Braconidae) parasitising adults in Europe. The normally hidden immature stages may be less vulnerable to parasitoid attacks.

Larvae of *P.attenuata* mine below-ground plant parts (Cox 2007, supplementary material) and may have been brought to North America with propagative material of hops. They may also have been spread rapidly within Canada by the same pathway. The distribution of *P.attenuata* also matches prior observations by Douglas et al. (2021) that most of the accidentally established non-indigenous Chrysomelidae in North America are European weed-associated species that have established in eastern Canada and north-eastern USA. It is also possible that *P.attenuata* was accidentally introduced into North America with weeds from family Cannabaceae transported with woody ornamental plants and soil during 1960 to 1965 as hypothesised by Douglas et al. (2021) for several other European Chrysomelidae. If they arrived with plants with soil during this period, then North American populations have been present for more than 50 years and their expansion may have been been gradual. Under this scenario, we would expect that *P.attenuata* is already present in north-eastern USA.

This new North American record, added to recent species counts (Douglas et al. 2021, Douglas et al. 2023) indicate that Canada and the USA are together known to host between 71 and 81 species of adventive Chrysomelidae. Of these, 54 to 62 adventive species of Chrysomelidae are known from Canada and 57 to 67 are known from the USA. This is the seventh or eighth adventive species of *Psylliodes* established in North America, with nearly a third of all European species already accidentally introduced into North America. *Psylliodeschalcomerus* (Illiger, 1807) was also intentionally introduced, perhaps unsuccessfully, as a biological control agent against adventive weeds. This is the 30^th^ to 35^th^ adventive member of subfamily Alticinae established in Canada and USA, of which 11 were introduced intentionally as biological control agents (Douglas et al. 2021). This reality, including several newly-recorded species in the past decade, suggests that additional European flea beetle species (and foliage-feeding leaf beetles more generally) will be detected as adventive in North America.

## Supplementary Material

XML Treatment for Psylliodes (Psylliodes) attenuata

## Figures and Tables

**Figure 1a.**
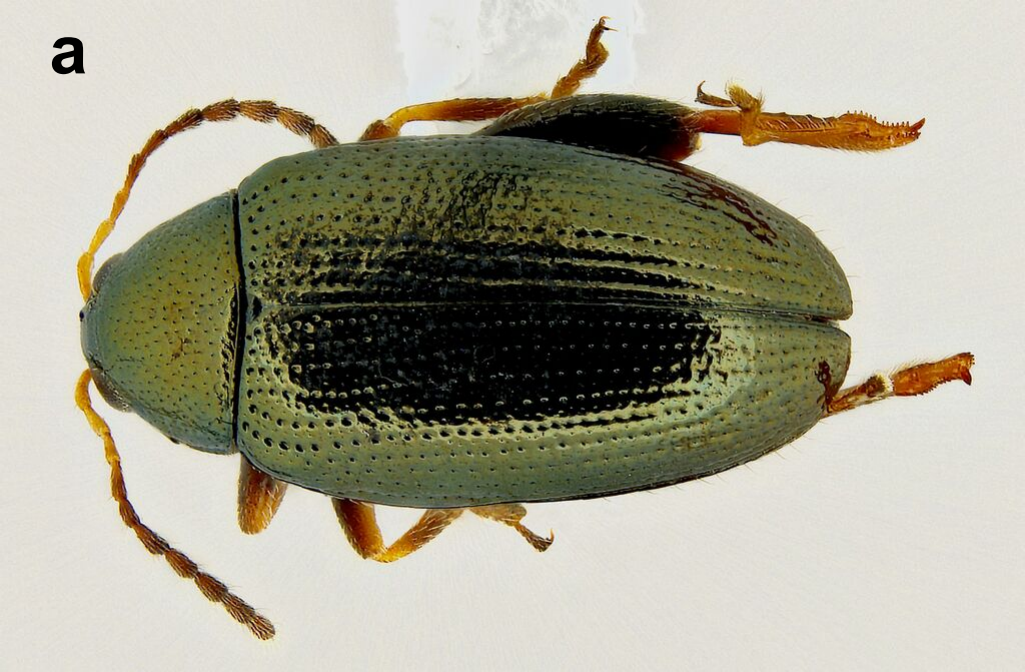
Dorsal habitus;

**Figure 1b.**
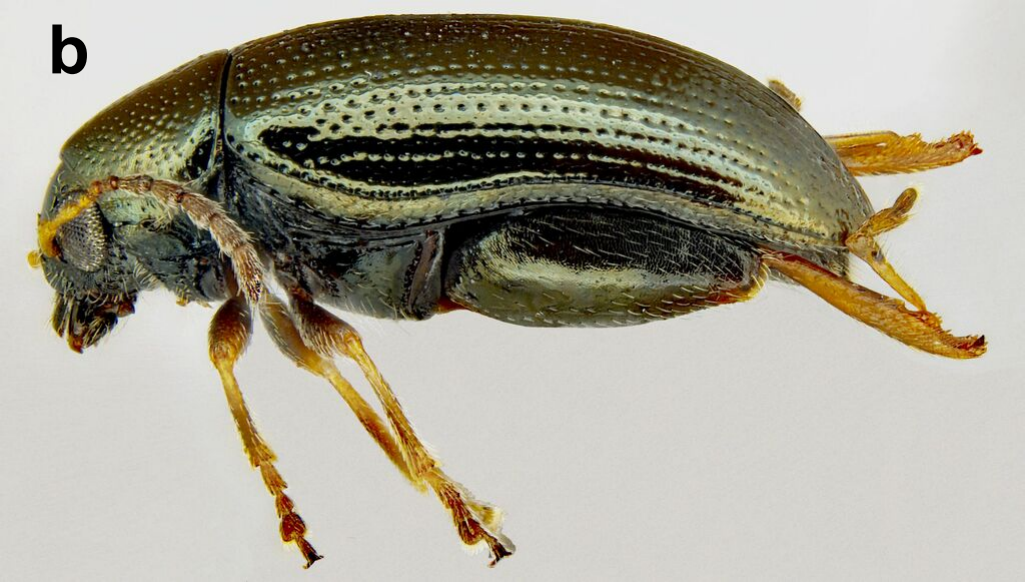
Lateral habitus;

**Figure 1c.**
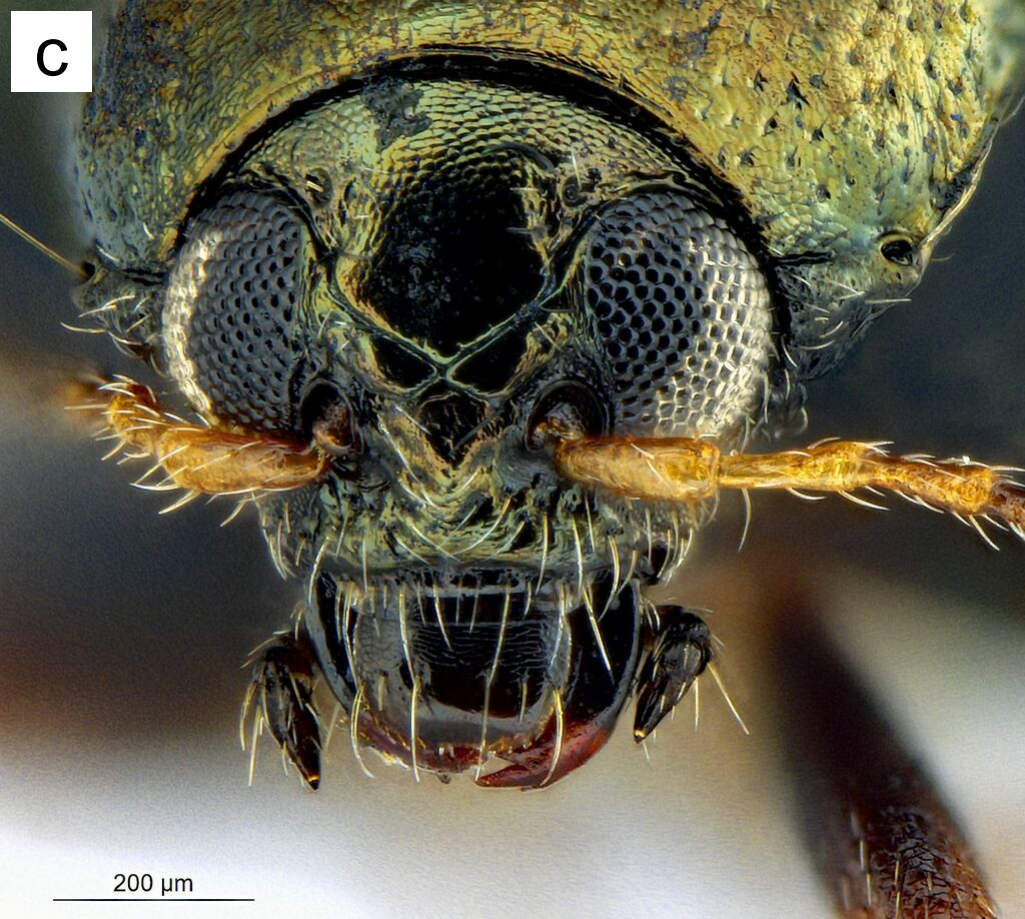
Head, frontal view;

**Figure 1d.**
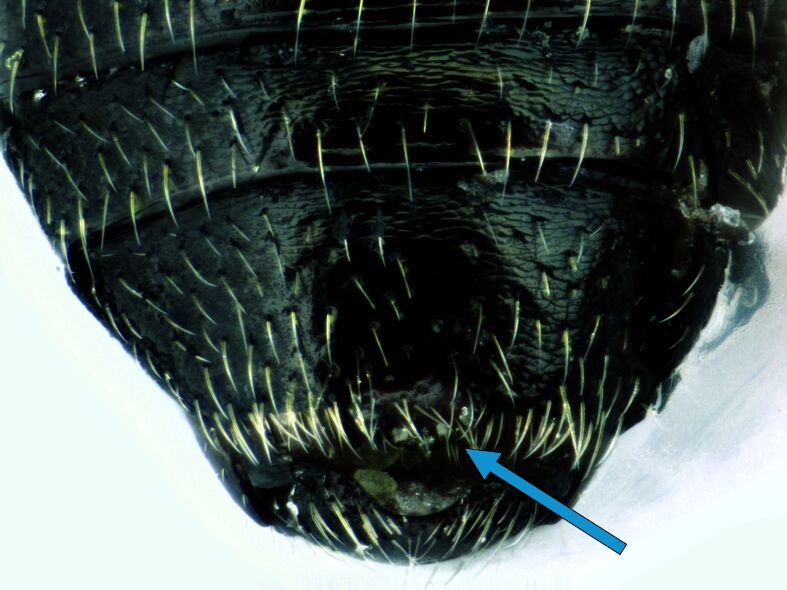
Abdominal ventrite V with arrow showing narrow posterior lobe;

**Figure 1e.**
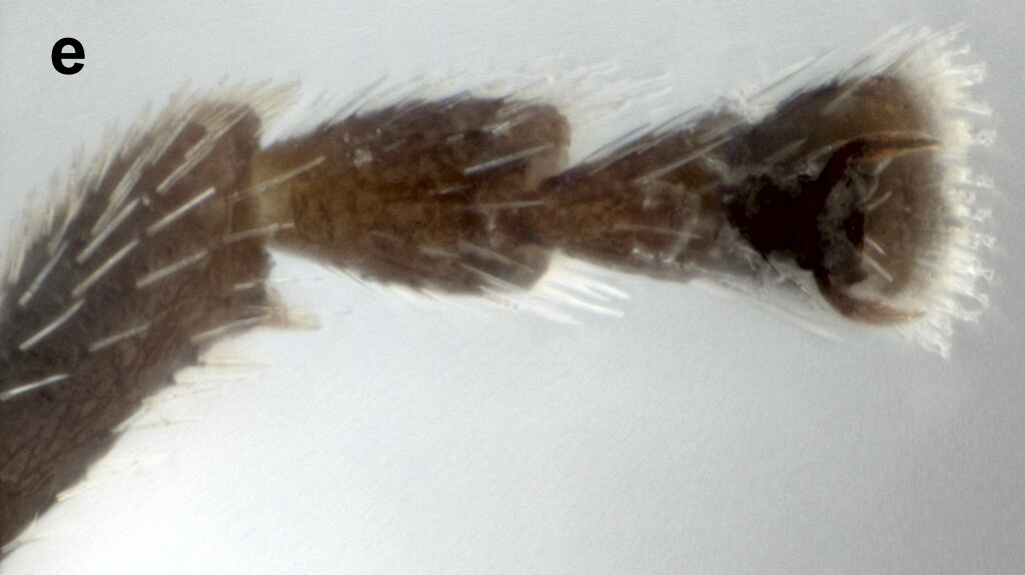
Tarsus of foreleg;

**Figure 1f.**
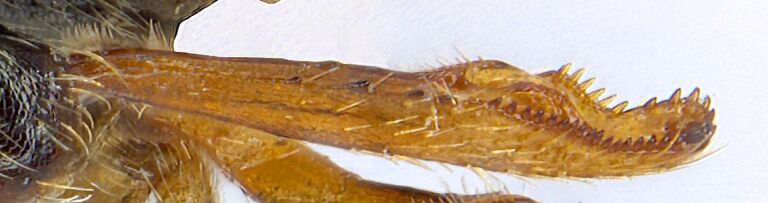
Hind tibia (a, b, c, f, M. Gamman AAFC; d, e, HBD).

**Figure 2a.**
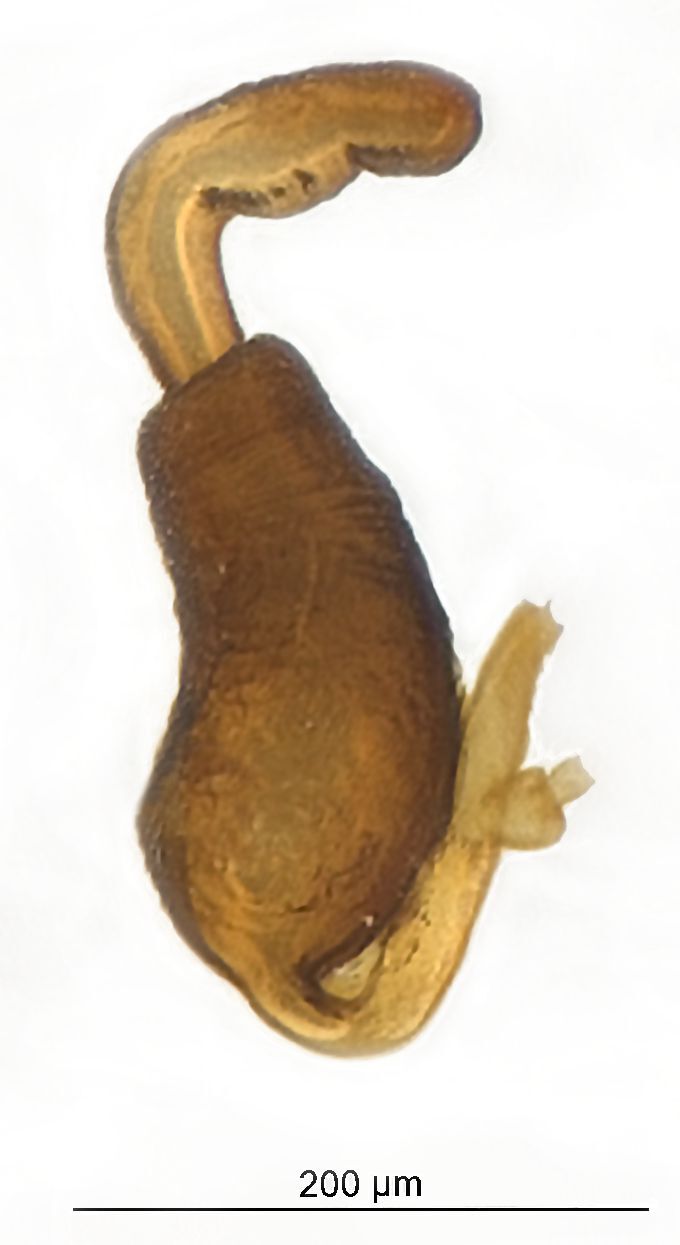
Female spermatheca;

**Figure 2b.**
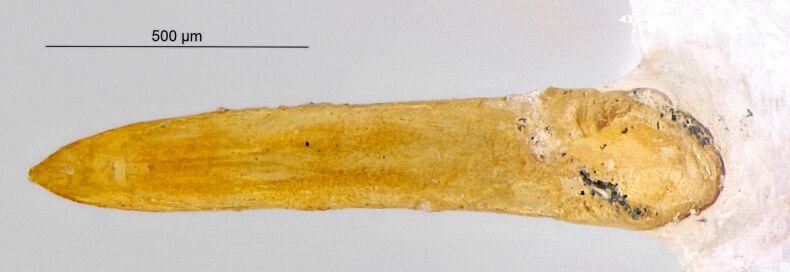
Male aedeagus, dorsal view;

**Figure 2c.**
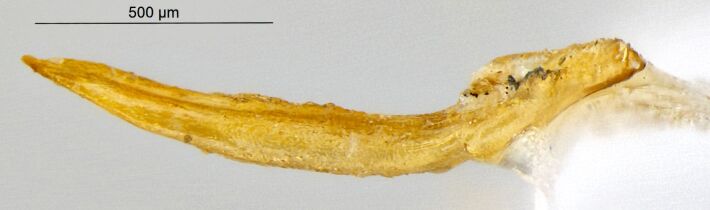
Aedeagus, lateral view (photos: M. Gamman, AAFC).

**Figure 4a.**
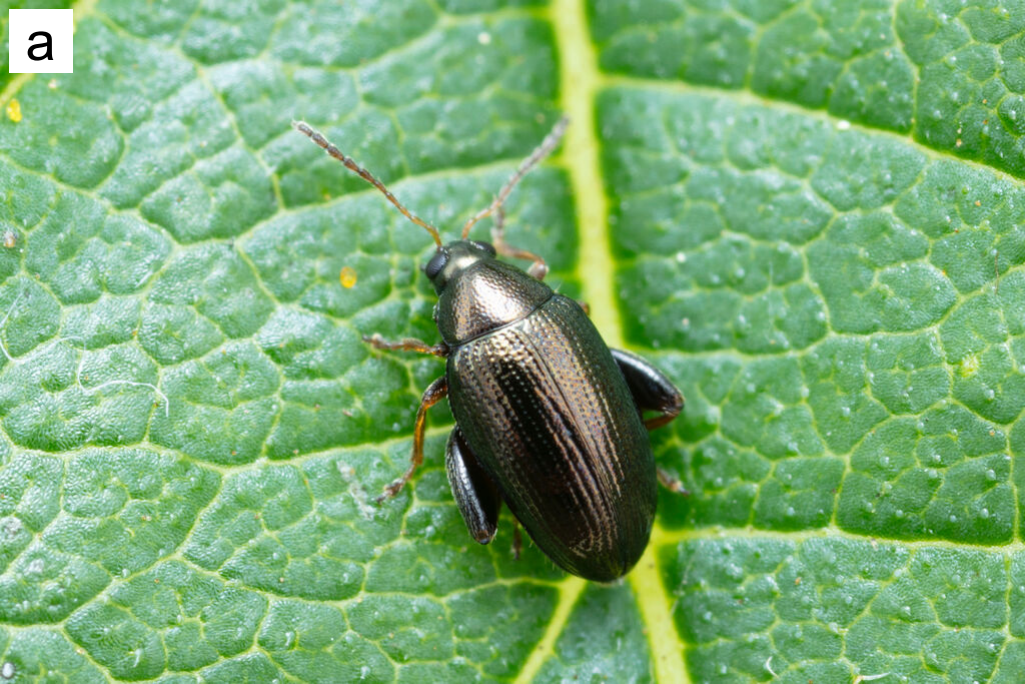
Adult in dorsal view;

**Figure 4b.**
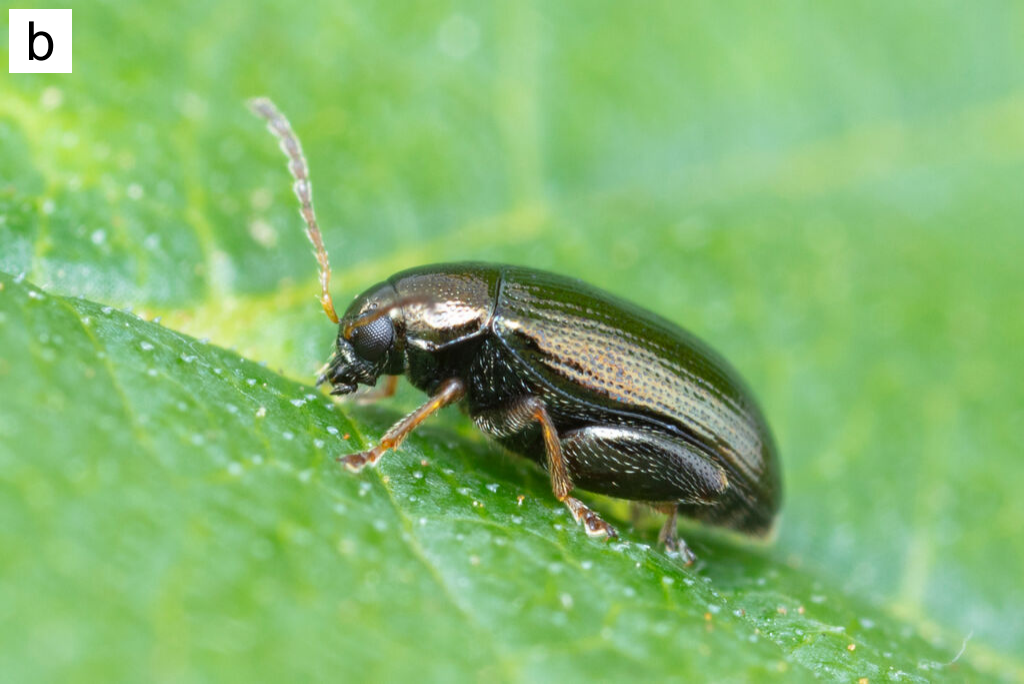
Adult in lateral view;

**Figure 4c.**
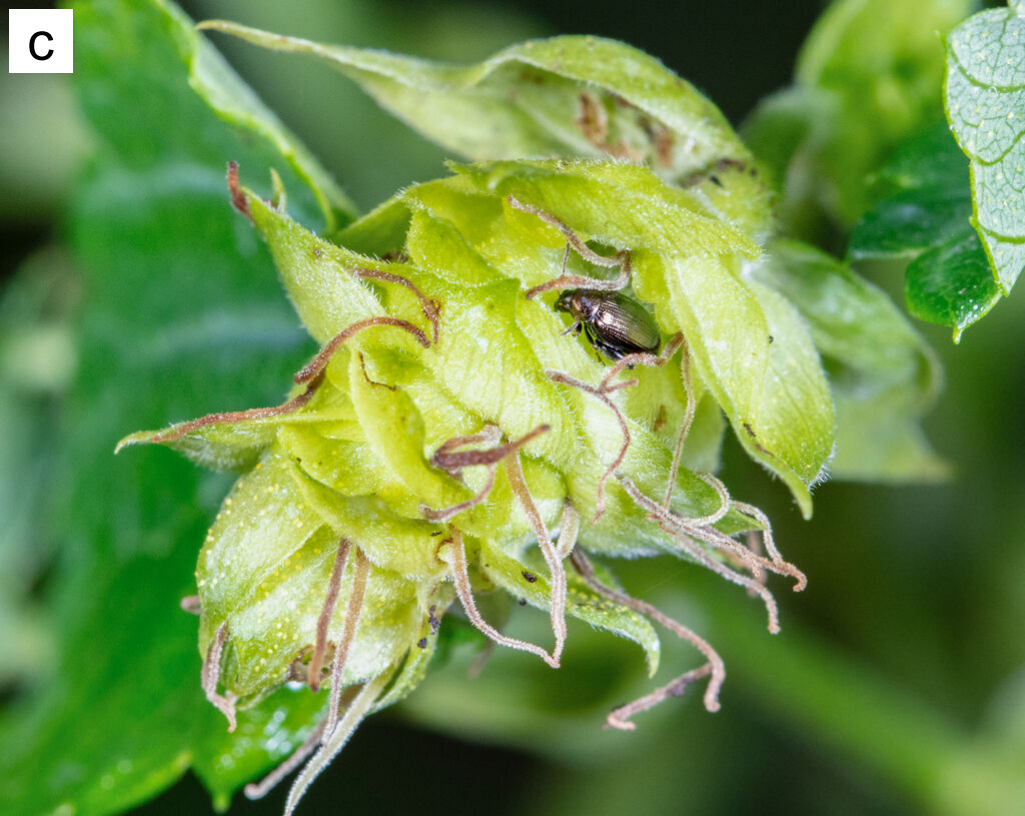
Adult inside hop cone;

**Figure 4d.**
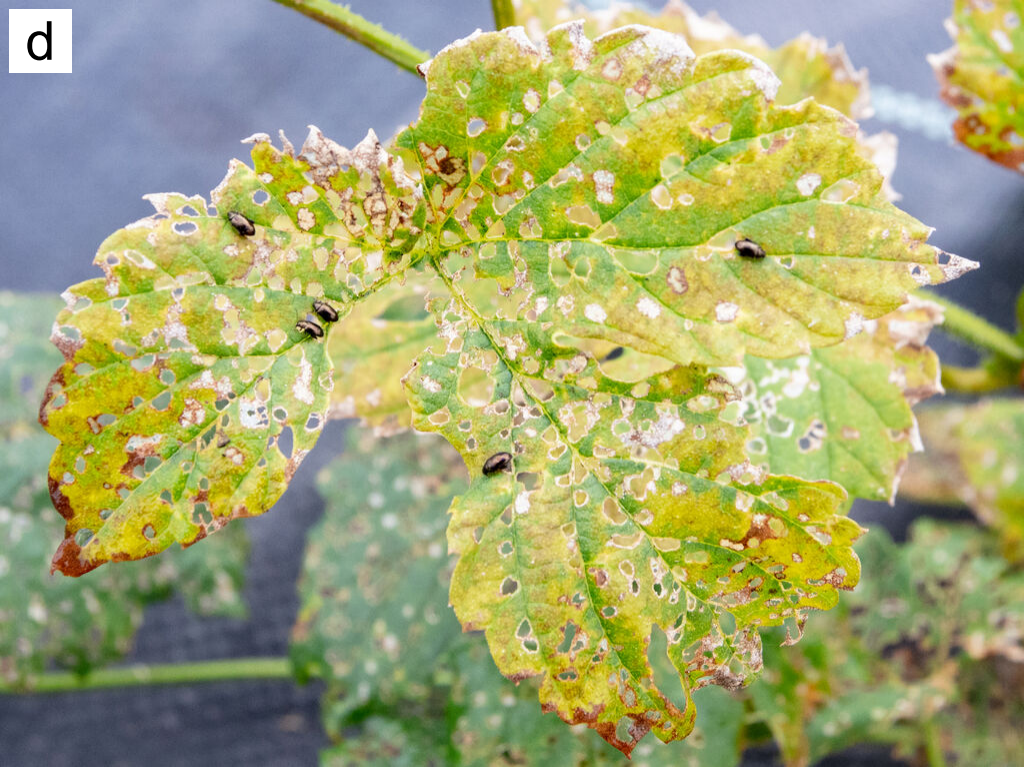
Damage to hop foliage;

**Figure 4e.**
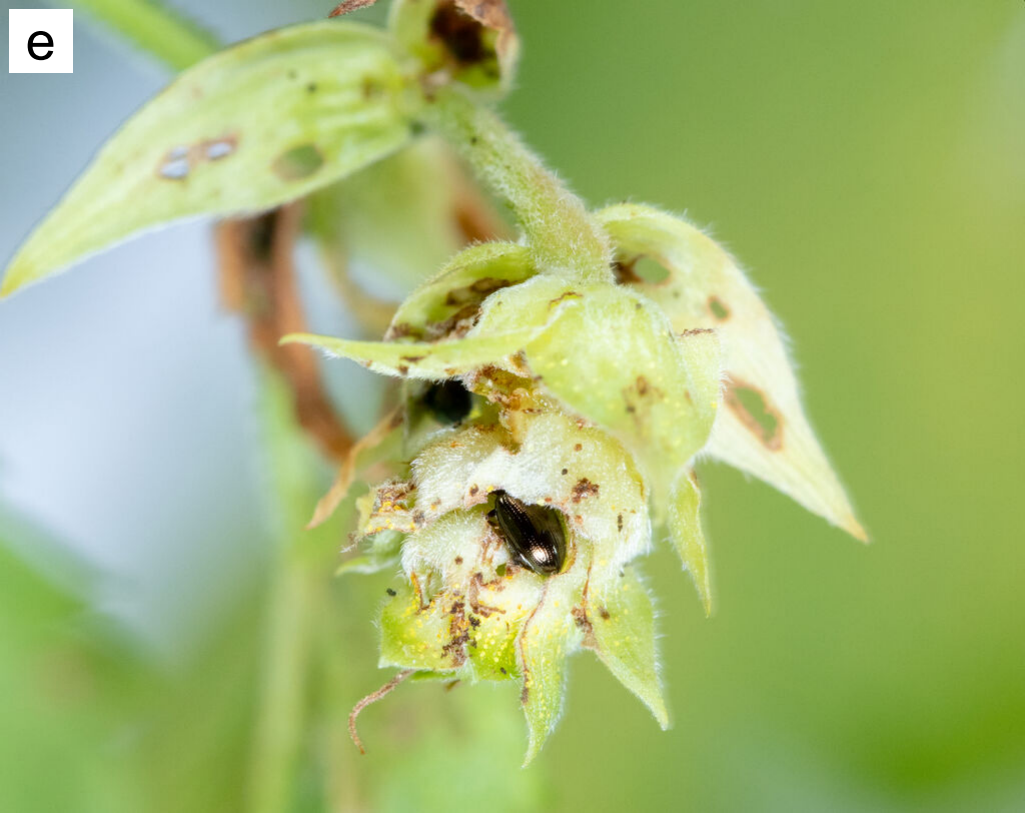
Damage to hop cone;

**Figure 4f.**
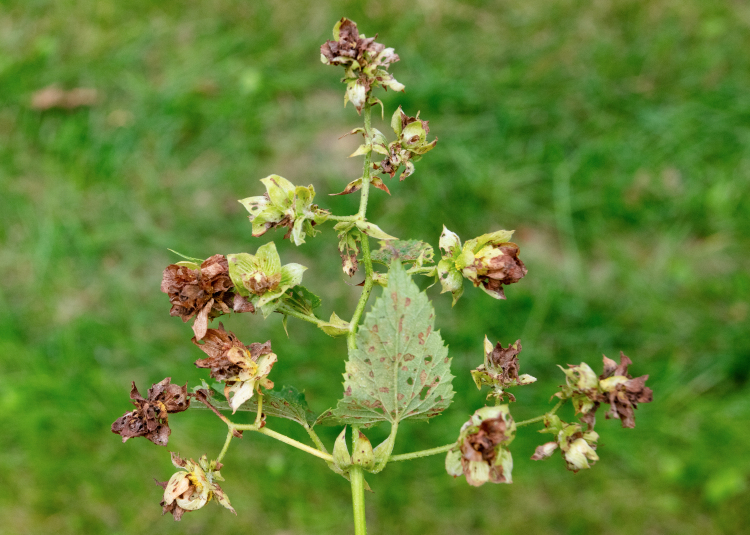
Damage to lateral shoot and cones (photos: JM-D).
